# Background predation risk induces anxiety-like behaviour and predator neophobia in zebrafish

**DOI:** 10.1007/s10071-024-01908-z

**Published:** 2024-10-23

**Authors:** Himal Thapa, Arash Salahinejad, Adam L. Crane, Ahmad Ghobeishavi, Maud C. O. Ferrari

**Affiliations:** 1 Department of Biology, University of Saskatchewan, Saskatoon, SK Canada; 2 Department of Veterinary Biomedical Sciences, WCVM, University of Saskatchewan, Saskatoon, Canada; 3 School of Mathematical and Natural Sciences, University of Arkansas, Fayetteville, US

**Keywords:** Alarm cues, Novel tank, Behaviour plasticity, Novel odour, Uncertainty

## Abstract

Prey face a major challenge in balancing predator avoidance with other essential activities. In environments with high risk, prey may exhibit neophobia (fear of novelty) due to the increased likelihood of novel stimuli being dangerous. The zebrafish, *Danio rerio*, is an established model organism for many scientific studies. Although spatial and object neophobia in zebrafish have received previous attention, little is known about the role of background risk in inducing neophobia in zebrafish. Here, we present two experiments using zebrafish to explore whether background predation risk can induce anxiety-like behaviour in a novel environment and neophobic responses when exposed to a novel odour. Over five days, we repeatedly exposed zebrafish to either high background risk in the form of chemical alarm cues (i.e., injured conspecific cues that indicate a predator attack) or a low-risk water control stimulus. In Experiment 1, when tested in a novel tank, zebrafish exposed to high predation risk displayed anxiety-like responses (reduced activity and increased bottom time spent) compared to their low-risk counterparts. Moreover, high-risk individuals showed reduced intra-session habituation to the novel tank compared to low-risk individuals. In Experiment 2, high-risk individuals exhibited fear responses toward a novel odour, unlike low-risk individuals. These results reveal that short-term repeated exposures to high risk can induce anxiety-like behaviour and predator odour neophobia in zebrafish.

**Supplementary Information:**

The online version contains supplementary material available at 10.1007/s10071-024-01908-z.

## Introduction

Balancing predator avoidance with other essential activities, such as foraging, courtship, and territorial defense, is a constant challenge for prey animals (Lima and Dill 1990; Sih 1992). Navigating these trade-offs is crucial due to the unforgiving nature of predation, as a misjudgment can cost the prey its life (Johnson et al. 2013; Lima and Dill 1990). Behavioural plasticity allows prey to exhibit appropriate antipredator responses depending on the level of predation threat (Brown et al. 2006; Helfman and Winkelman 1997). Such plasticity enables prey to balance predator avoidance and other fitness-related activities (Brown 2003). For example, small reef fishes exhibited suppressed foraging behavior near potential predators (Catano et al. 2016). Individuals displaying suitable antipredator responses toward predators are more likely to survive (Blumstein et al. 2002).

To respond to predators, prey must first be able to sense danger (Endler 1986). Prey depend on accessible public information (tactile, auditory, visual, or chemical) regarding local threats to perceive risk (Bouskila and Blumstein 1992; Chivers and Smith 1998; Endler 1986). Many aquatic species use chemical alarm cues as a reliable source to perceive predation risk. Chemical alarm cues are substances released from prey tissue when a predator damages it during an attack (Ferrari et al. 2010). When nearby individuals detect conspecific alarm cues, they exhibit overt antipredator responses (e.g., in zebrafish; Attaran et al. 2019 ). In general, the intensity of such antipredator responses is known to match the intensity of the threat (i.e., threat-sensitivity)(Helfman 1989).

According to the “dangerous niche hypothesis”, individuals from high-predation environments should exhibit strong anti-predator responses to novel cues, as novelty is likely dangerous (Greenberg 2003). Such responses to a novel cue can be driven by instinct, personality traits, and as the result of decision based on existing sensory cues influenced by prior experience. Such fear of novelty (i.e., neophobia) should increase the probability of survival if novel cues are indeed dangerous (Crane et al. 2020). Prey have been reported to show phenotypically-plastic neophobia toward cues such as odours from novel species that are potential predators (i.e., predator neophobia), as well as fear towards a novel environment where danger might be present (i.e., spatial neophobia) (Brown et al. 2013; Crane and Ferrari 2017). For example, juvenile convict cichlids, *Amatitlania nigrofasciata*, and wood frog tadpoles, *Lithobates sylvaticus*, that were repeatedly exposed to conspecific alarm cues over a period of a few days became neophobic toward odours from novel species (Brown et al. 2013). Similarly, in fathead minnows, *Pimephales promelas*, repeated exposures to conspecific alarm cues induced spatial neophobia (Crane et al. 2020). In the presence of an actual threat, such neophobia can decrease the chances of making costly decisions until more data is obtained (Elvidge et al. 2016). Hence, neophobia allows prey to increase their survival in encounters with a novel predator, as has been found in whitetail damselfish, *Pomacentrus chrysurus*, (Ferrari et al. 2015) and wood frog tadpoles (Crane et al. 2018). However, when a novel cue is not dangerous, showing generalized fear responses can be costly (Crane et al. 2020).

Habituation is a simple associative learning mechanism that allows organisms to adjust to novel environments by reducing their anxiety responses over time (Bolivar 2009; Hemmi and Merkle 2009; Leussis and Bolivar 2006; Thompson and Spencer 1966). It can reflect different types of memory: inter-session habituation is often used to assess mid- to long-term spatial memory, while intra-session habituation indicates spatial working memory (Müller et al. 1994). The ability to habituate can be adaptive and maladaptive, depending on the context. In low-risk environments, individuals with faster habituation may have higher foraging success. However, in high-risk environments, faster habituation can be risky, as it may increase the likelihood of encountering predators. Previous studies have reported that background level of risk favored learning and retention of predator-related information (Chivers et al. 2014; Ferrari et al. 2015; Joyce et al. 2016; Mitchell et al. 2016). For example, high-risk wood frog tadpoles, *L. sylvaticus*, showed higher antipredator responses toward a learned predator odour and retained the information for extended periods. Studies on how background levels of risk influence habituation are scarce.

Zebrafish, *Danio rerio*, has been widely used as a model species across biological fields, including genetics, pharmacology, neuroscience, and developmental biology (Miller and Gerlai 2011; Petersen et al. 2022). Characteristics such as their easy maintenance in high density, ability to produce many offspring, and high genetic homology with humans have made zebrafish an ideal model organism for such research (Barbazuk et al. 2000; Lawrence 2007). While zebrafish have gained popularity as a model for behavioural studies including anxiety-like behaviours (Barcellos et al. 2010; Gerlai 2010; Krook et al. 2019; Miller and Gerlai 2011; Orger and Polavieja 2017), neophobia in zebrafish had not been investigated until recently (Franks et al. 2022; Lucon-Xiccato et al. 2020; Quadros et al. 2019; Roy et al. 2017; Scatterty and Hamilton 2024). To our knowledge, only one study has explored conspecific alarm cue induced anxiety-like behaviours in zebrafish (Quadros et al. 2019). Following repeated exposures to alarm cues over seven days, two zebrafish populations (*wild-type* and *leopard*) showed anxiety-like behaviours in a novel tank (i.e., spatial neophobia) (Quadros et al. 2019). However, it is still unknown whether repeated exposures over few days would induce neophobia in zebrafish in response to a novel predator odour (i.e. predator neophobia). In this study, we performed two experiments to investigate the role of background risk in inducing anxiety-like behaviours in a novel tank and predator odour neophobia in zebrafish. For both experiments, we repeatedly exposed the zebrafish to either alarm cues to simulate a high-risk environment or a water control (low-risk) over five days. One day later, we tested zebrafish anxiety-like behaviours in a novel tank (Experiment 1) and fear responses in the presence of a novel predator odour (rainbow trout, *Oncorhynchus mykiss*) (Experiment 2). We hypothesized that high background risk would induce anxiogenic behaviour and predator neophobia in zebrafish, as a previous study reported that zebrafish exhibit anxiety-like behaviour in a novel tank following repeated exposure to risk (Quadros et al. 2019). Hence, we predicted that high-risk zebrafish would be less active and exploratory in the novel tank and when exposed to the novel odour. We also hypothesized that background risk exposures may influence the rate of habituation in a novel tank. We predicted that the low-risk individuals would show less anxiety-like behaviours compared to the high-risk individuals between intra-session observations in the novel tank.

## Materials and methods

### Test species and maintenance

We used 150 experimentally naïve wild-type (AB) adult zebrafish (originated from colonies at the University of Oregon, ~ 50:50 male and female, 12 months old) from a stock colony housed at the R. J. F. Smith Centre for Aquatic Ecology at the University of Saskatchewan. Zebrafish were transferred into 25 tanks (2.8 l tank, six fish per tank) with flow-through water that was filtered and dechlorinated. This water (hereafter, ‘facility water’) had a total hardness of 150 mg/l, alkalinity of 120 mg/l, pH of 7.6-8, and a temperature of 24-27^o^ C. All sides of the tank except the front were covered with opaque plastic sheets to block visual stimuli from nearby tanks. We fed zebrafish twice daily (07.00 h and 17.00 h) with a commercial flake food (Nutrafin Max flakes, Holm, Germany).

To obtain a novel odour, we used four rainbow trout (fork length 15.0–18.5 cm) from a stock colony at the Toxicology Centre at the University of Saskatchewan. The trout were housed in a 600 l flow-through pool with facility water. We fed the rainbow trout daily with commercial trout pellets. All fish were provided with a 14:10 light-to-dark cycle.

### Cue collection

#### Alarm cues

Following standard methods (Attaran et al. 2019), we obtained zebrafish alarm cues by euthanizing five donor individuals with a blow to the head followed by a collection of 5.74 cm^2^ skin from the lateral sides of the body. We homogenized the skin in 200 ml of facility water and removed any remaining large particles by filtering the solution through a mesh (0.5 mm). The resulting solution was then diluted with water to reach a final concentration of -1 cm^2^ per 40 l of water, which is known to elicit a significant antipredator response in zebrafish (Attaran et al. 2019). We then stored the final solution in 100 ml aliquots at 20^o^C until it was thawed and used for the exposure treatment. Additionally, we froze 20 ml samples of facility water as a control.

#### Novel odour

To obtain a novel odour, we transferred the trouts into individual 38 l tanks filled with clean facility water at a volume standardized for the size of the fish (50 ml/g of fish). The fish had been deprived of food for 48 h beforehand to minimize diet cues (Ferrari et al. 2008; Scherer and Smee 2016). After 24 h, tank water was collected and frozen in 600 ml aliquots until being thawed before use.

### Experimental overview

#### Background risk exposure phase

We exposed zebrafish in 2.8 l plastic tanks equipped with an air stone. In Experiment 1, we exposed 5 groups of six individuals per treatment (two treatments, 10 tanks). In Experiment 2, we exposed 7 groups of six individuals for high-risk exposure and 7 groups of five to six individuals (4 groups had six individuals and 3 groups had five individuals) for low-risk exposure. All sides of the tank except the front were covered with opaque plastic sheets to block visual stimuli from nearby tanks. A 150 cm injection hose, attached parallel to the air stone, facilitated the gentle introduction of 0.5 ml of alarm cues (high risk) or facility water (low risk) into the tank using a syringe (Crane et al. 2015; Ferrari and Chivers 2006a, b). Injections occurred three times a day for five days, once in the morning (08.00–11.00 h), at midday (11.00–13.00 h) and in the afternoon (13.00–16.00 h). Most of the previous studies used 12 to 14 exposures over four to seven days to induce neophobia in wild-caught fish (Brown et al. 2013; Chivers et al. 2014; Crane et al. 2015). Since the zebrafish individuals have been lab bred and lab reared for multiple generations, we selected 15 exposures over five days for a successful background exposure phase. Also, we randomized the time of injections so that individuals do not associate risk with any specific time of the day (Chivers et al. 2014). A complete water change was only conducted one hour after the third exposure each day.

#### Novel tank test (experiment 1)

We assessed anxiety-like behaviour in a novel environment 24 h after the background exposure phase. This ‘novel tank test’ is an established paradigm to study anxiety (Blaser and Rosemberg 2012; Cachat et al. 2010, 2011; Salahinejad et al. 2022). The test tank differed in size (28.8 × 16.2 × 10 cm) and shape (rectangular) from the holding tanks. We used two 13 watt fluorescent bulbs to illuminate the tank from above. White plastic sheets covered all three sides of the tank except the front. Additionally, the tank was equipped with a plastic injection hose that ended at 1.5 cm below the water surface. We placed a single zebrafish into the test tank filled with tap water and acclimated it for 10 s. Following the acclimation, we recorded zebrafish behaviour for eight minutes using an HD webcam (C922x Pro Stream, Logitech, Lausanne, Switzerland). We used a short acclimation period to prevent individuals from becoming accustomed to the new tank. We tested a total of 60 individuals.

#### Predator odour neophobia (experiment 2)

To assess the response to a novel predator odour, we subjected zebrafish to the background risk exposure phase mentioned above, followed by testing individual behaviour in response to the novel predator odour (rainbow trout odour). One day after the last alarm cues exposure, we placed a single zebrafish in the novel tank for 30 min to allow it to habituate to the new environment. Previous studies have reported that a 30-minute period is enough for zebrafish to habituate in a novel tank (Raymond et al. 2012; Wong et al. 2010). We then began the predator neophobia testing trial by recording zebrafish in the tank for six minutes (i.e., the ‘pre-stimulus period’). Following the pre-stimulus period, we gently injected 10 ml of rainbow trout odour or facility water (control) into the tank and recorded individual behaviour for another six minutes (the ‘post-stimulus period’). We tested a total of 80 individuals in this experiment.

#### Quantification of behaviour

We analyzed the recorded videos using Ethovision XT (Noldus Info Tech., Wageningen, The Netherlands) as described by (Blaser and Gerlai 2006; Gerlai et al. 2008). Briefly, we used automatic tracking by the software to identify the subject within a defined arena (test tank area) at 10 Hz recording frequency (once every 0.1 s). As a result, 600 position x, y coordinate pairs per minute were recorded for each fish. Using the software, we defined three equal vertical zone (bottom, middle, and top) on the testing tank. We quantified the distance travelled, time spent in each zone, and latency to reach the top layer. Previous studies have reported that fearful zebrafish show reduced movement, more time spent in the bottom layer of the tank, less time spent in the top layer, and a longer latency to reach the top layer (Blaser and Rosemberg 2012; Gerlai et al. 2000; Jesuthasan and Mathuru 2008; Pfeiffer 1977; Salahinejad et al. 2022).

### Statistical analysis

#### Novel tank test (experiment 1)

We performed a principal component analysis (PCA) to reduce the number of response variables by creating a composite anxiety response using the variables: total distance travelled, time spent in the bottom zone, and latency to reach the top zoner of the tank. The PCA used a covariance matrix. For the novel tank test, the PCA resulted in one axis (PC1) that explained 82.06% of the total variance (eigenvalue > 2). The scores loaded heavily on less distance traveled (-0.95), more time spent in the bottom zone (-0.94), and a longer latency to reach the top zone (0.82). Hence, we refer to PC1 as the ‘anxiety responses’ hereafter. We then analyzed differences in PC1 between high- and low-risk zebrafish using a Type-I nested two-way ANOVA, with the background risk treatment, and sex as fixed factors and the exposure tank as a random factor, with fish nested within their exposure tanks. We therefore considered the tank, rather than the fish, the unit of replication.

#### First 4 min. vs. last 4 min. In the novel tank

To examine the change in anxiety responses over time in the novel tank, we compared individual behaviours between the first 4 min. and the last 4 min. for both the high-risk and low-risk groups. For this analysis, we considered the total distance traveled and time spent in the bottom zone. We avoided using the latency to top zone behaviour for this analysis. We performed a two-way ANOVA for each behavioural parameter, with background risk (high-risk vs. low-risk) and sex (male vs. female) as fixed factors.

#### Predator odour neophobia (experiment 2)

##### Novel odour baseline test

We did a PCA to reduce the number of variables to analyze the pre-stimulus baseline behaviour (i.e., before the novel odour was introduced). PC1 explained 57.33% of the total variance (eigenvalue ~ 2) and loaded heavily on less distance travelled (-0.59), more time spent in the bottom zone (0.81), and a longer latency to reach the top zone (0.84) (Fig. 2). We analyzed this ‘baseline fear response’ (PC1) using a Type-I three-way nested ANOVA with the background risk treatment (high or low), the test cue (novel odour or water), sex (male or female), and their interaction as fixed factors and the exposure tank as a random factor. This confirmed that the pre-stimulus fear response did not differ significantly across the treatments (all *P*’s > 0.05).

##### Novel odour response

As zebrafish had similar baseline activity, we calculated the change (post-stimulus – pre-stimulus) in behavioural variables (distance travelled, time in the bottom zone, and latency to reach the top zone) and included these variables in a PCA. PC1 explained 73.11% of the variance (eigenvalue ~ 2) and loaded on loaded heavily on less distance travelled (-0.74), more time spent in the bottom zone (0.86), and a longer latency to reach the top zone (0.93). We then analyzed PC1 (i.e., the ‘change in fear response’) with a Type-I three-way nested ANOVA, including the background treatment (high risk or low risk), the test cue (novel odour or water), sex (male or female), and their interaction as fixed factors, and exposure tank as a random factor. To further explore the interaction term, we split the data by the background treatment and used separate independent t-tests to compare responses to the test cues.

##### First 3 min. vs. last 3 min. During the post-stimulus period

To examine the change in fear responses during the post-stimulus period following the novel odour exposure, we compared individual behaviours between the first 3 min and the last 3 min of the post-stimulus period. We only considered the high-risk individuals exposed to the novel odour, as we found that only high-risk individuals showed significant behavioural responses following its exposure (see result below). For each behavioural parameter, we performed an independent t-test between the first 3 min and the last 3 min observation. All analyses were conducted in SPSS 26.0 with α = 0.05.

## Results

### Novel tank test (experiment 1)

**Fig. 1:**
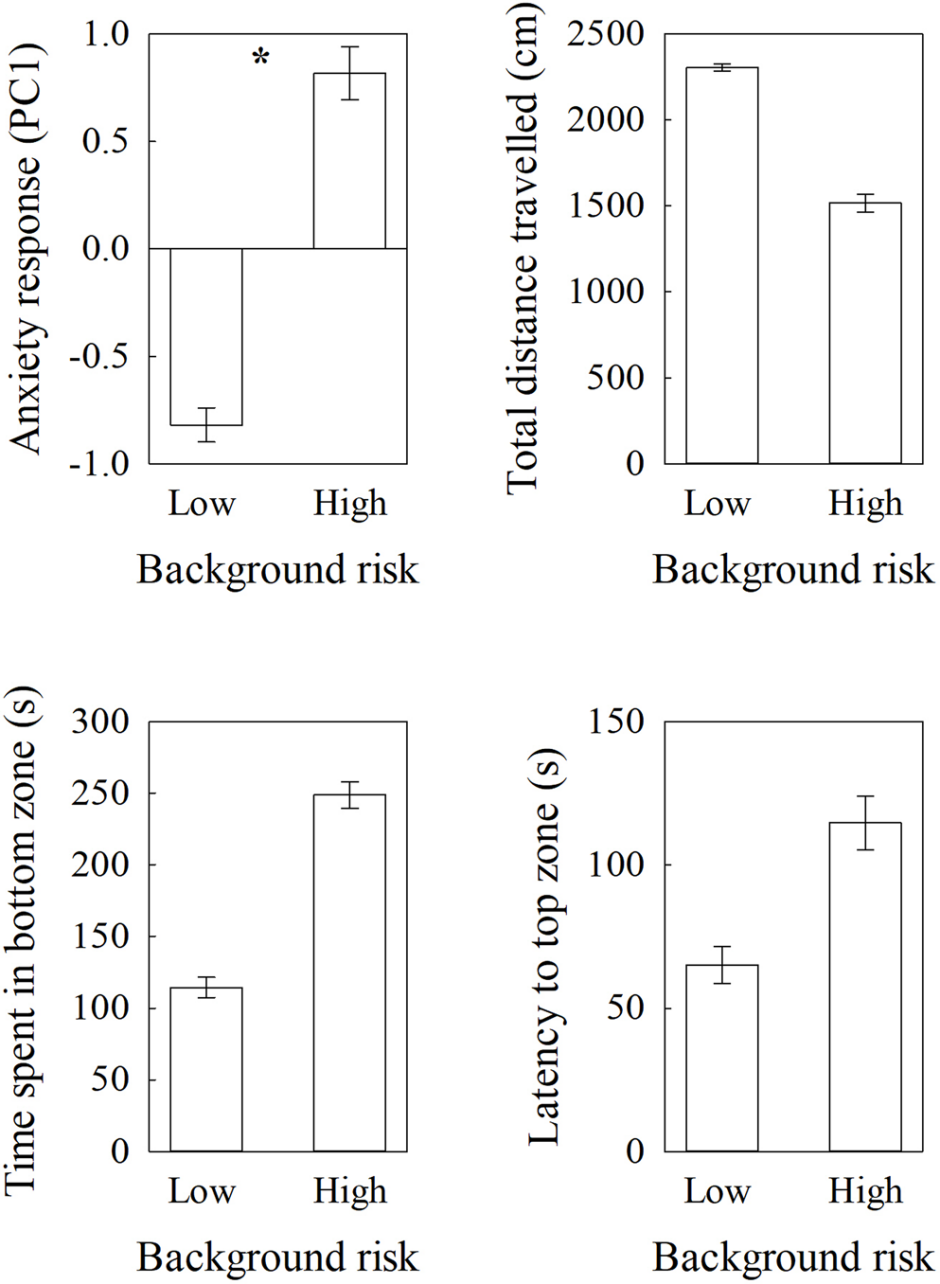
Mean (± SE) **a**) anxiety response (PC1), **b**) total distance travelled, **c**) Time spent in bottom zone, and **d**) latency to top zone, in a novel tank by zebrafish from either a high-risk (alarm cues) or low-risk (water) background

We found a significant effect of background risk on fear responses of zebrafish in the novel tank, where individuals with repeated alarm cues exposure (high-risk) showed significantly higher anxiety responses compared to individuals exposed to water (low-risk) (*F*_1,7.4_ = 730.24, *P* < 0.001; Fig. 1). We did not find an effect of sex (sex: *F*_1,48.67_ = 0.09, *P* = 0.77; sex x risk: *F*_1,48_ = 0.13, *P* = 0.72). We also did not find a significant effect of exposure tank on the anxiety response (*F*_8,48_ = 0.15, *P* = 0.99). *First 4 min. vs. last 4 min. in the novel tank:*

**Fig. 2:**
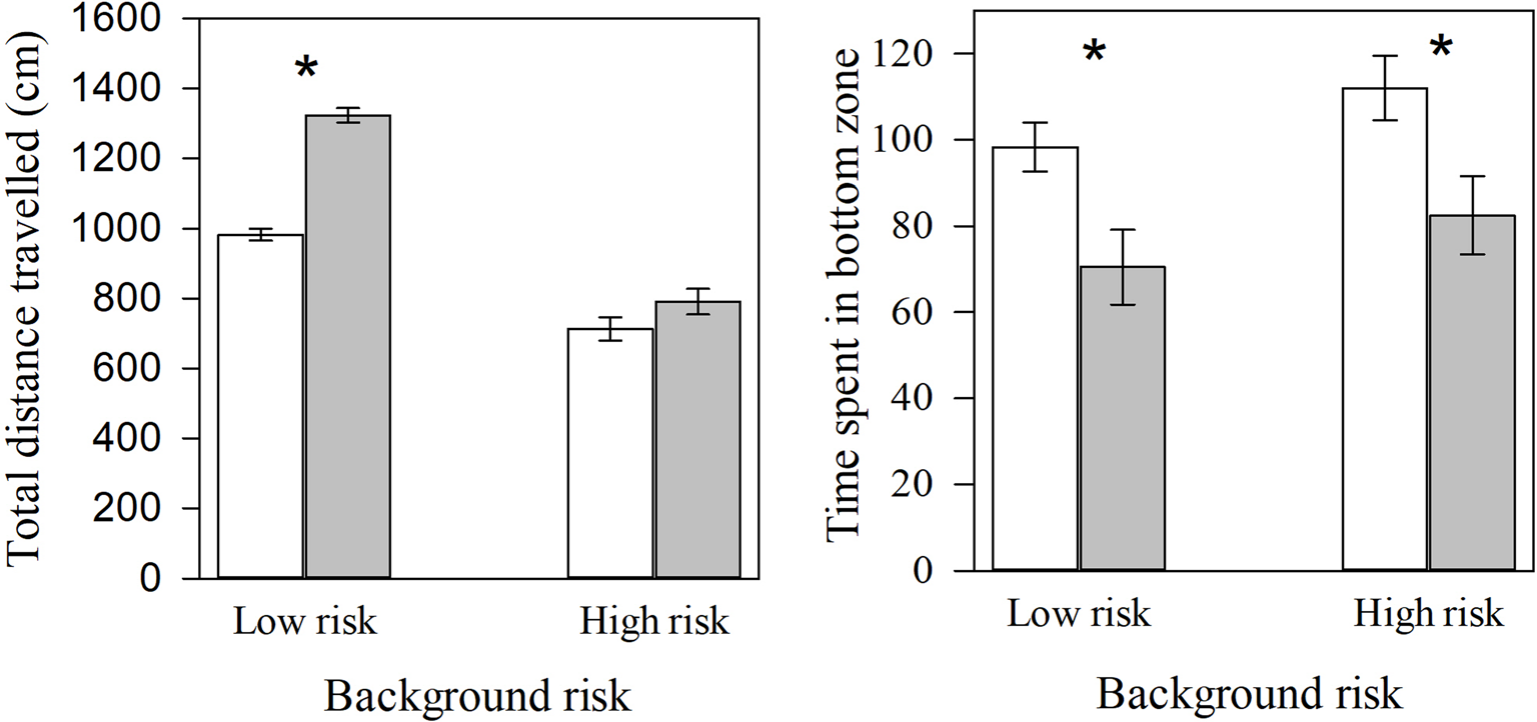
Mean (± SE) **a**) total distance travelled, and **b**) time spent in bottom zone between the first 4 min and the last 4 min in a novel tank by zebrafish from high-risk (alarm cues) or low-risk (water) background. White bars represent the first 4 min., and grey bars represent the last 4 min. of observation

We only found a significant interaction effect of background risk and observation period on zebrafish distance travelled (*F*_*1,112*_ = 21.20, *P* < 0.001, Fig 2a). Low-risk individuals showed a significant increase in distance travelled during the last 4 min. compared to the first 4 min. (t_58_ = -12.74, *P* <0.001, Fig. 2a). On the other hand, high-risk fish did not show any difference in distance travelled between the first 4 min. and the last 4 min. (t_58_ = -1.58, *P* = 0.12, Fig. 2a). On the other hand, both high-risk and low-risk individuals showed significant reduction in time spent in the bottom zone between during the last 4 min. compared to the first 4 min. (*F*_1,112_ = 12.97, *P* < 0.001, Fig, 2b).

### Predator neophobia (experiment 2)

**Fig. 3:**
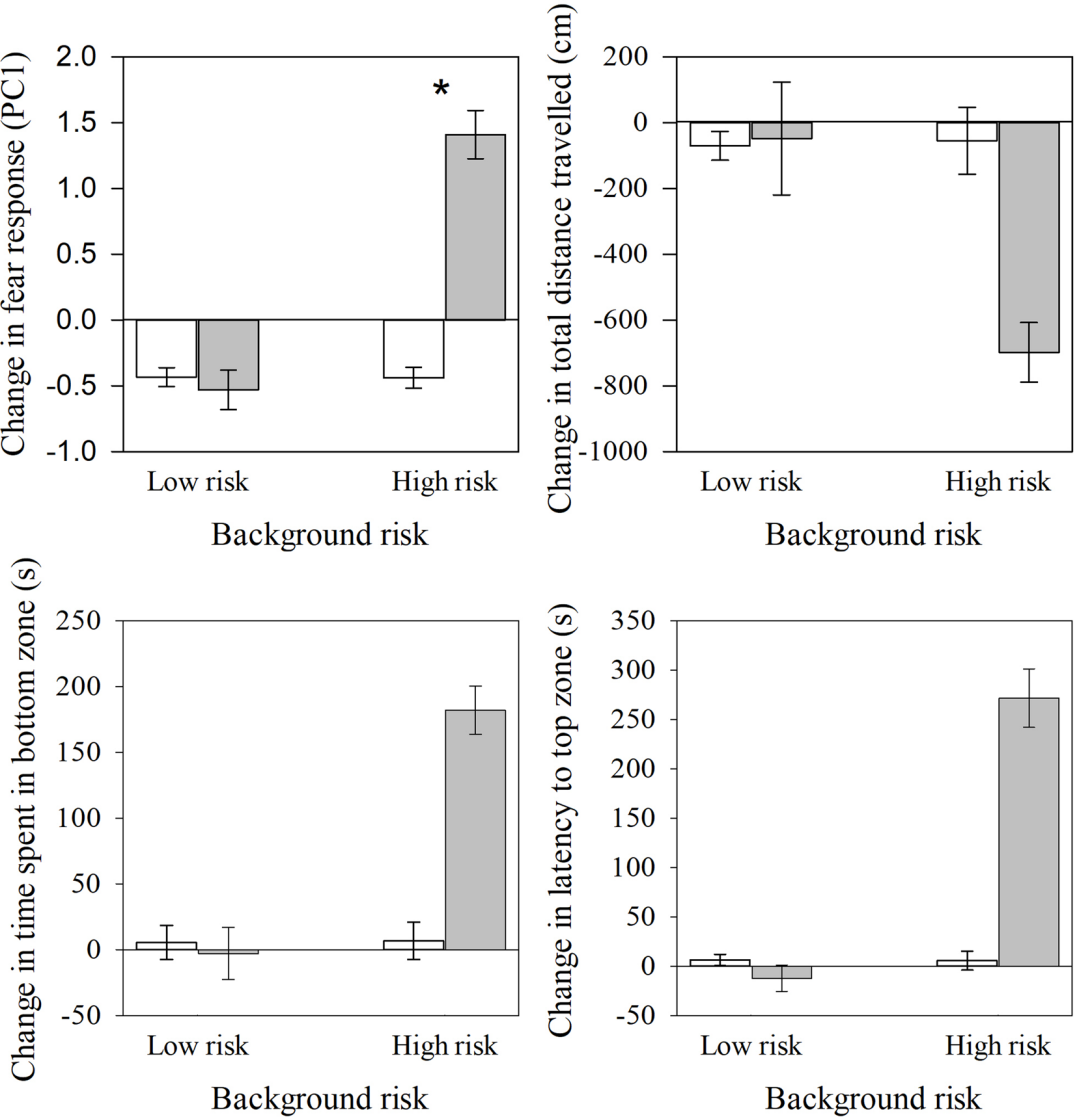
Mean (±SE) **a**) change in fear response (PC1), **b**) change in total distance travelled, **c**) change in time spent in bottom zone, and **d**) change in latency to top zone by zebrafish from high-risk (alarm cues) or low-risk (water) background when tested for a response to a novel odour vs. a water control. White bars represent water cue, and grey bars represent novel odour

We found a significant interaction effect of background risk and test cue on the change in fear responses of zebrafish (*F*_1,60_ = 56.46, *P* < 0.001; Fig. 2). Individuals from high background risk showed a significantly higher fear responses when exposed to a novel predator odour compared to water (*t*_25.77_ = -9.25, *P* < 0.001; Fig. 2 and 3). However, low-risk zebrafish showed no significant fear responses when exposed to a novel predator odour or water (*t*_38_ = 0.59, *P* = 0.56; Fig. 2). We did not find any other significant interaction effects (sex: *F*_1,71.07_ = 0.17, *P =* 0.68; sex × risk: *F*_1,60_ = 0.54, *P =* 0.47; sex × test cue: *F*_1,60_ = 0.01, *P =* 0.92). We did not find a significant effect of background conditioning tanks on the change in fear response (*F*_12,60_ = 1.71, *P* = 0.09).

### First 3 min. vs. last 3 min during post-stimulus period

**Fig. 4:**
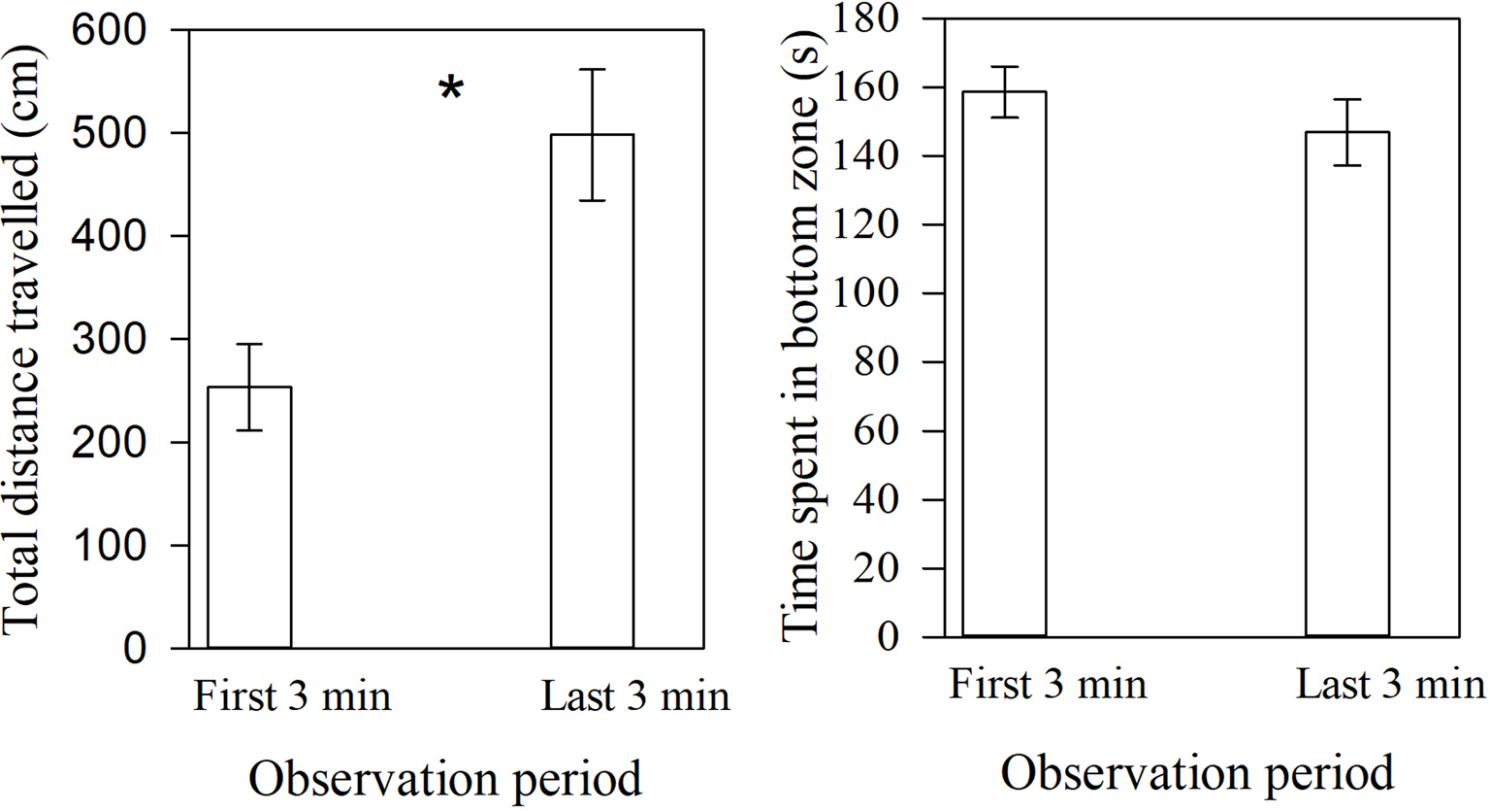
Mean (± SE) **a**) total distance travelled, and **b**) time spent in bottom zone by zebrafish from high-risk (alarm cues) background between the first 3 min and the last 3 min during the post-stimulus period when tested for a response to a novel odour

During the post-stimulus period, we found a significant increase in distance travelled between the first and last 4 min by high-risk individuals (*F*_1,36_ = 9.89, *P* = 0.003, Fig. 4a). However, individuals showed similar time spent in the bottom zone between the first and the last 3 min during the post-stimulus period (observation period: *F*_1,36_ = 0.90, *P* = 0.35; sex: *F*_1,36_ = 0.27, *P* = 0.61; observation period x sex: *F*_1,36_ = 0.27, P = 0.61 Fig 4b).

## Discussion

Our results suggest that repeated exposures to high predation risk can induce anxiety-like behaviour in a novel tank and predator odour neophobia in zebrafish. Consistent with the “dangerous niche hypothesis” (Greenberg and Mettke-Hofmann 2001; Mettke-Hofmann et al. 2013), high-risk zebrafish showed reduced distance moved, increased time spent in the bottom zone, and delayed entry into the top zone of the novel tank. Furthermore, high-risk individuals displayed higher fear responses toward a novel predator odour, showing adaptive behaviours in response to potential threats from an unknown predator. Previous studies have also reported predator neophobia in other fish species (Elvidge et al. 2016; Feyten et al. 2019). Our results are consistent with the finding of Quadros et al. (2019), who reported anxiety-like behavior in zebrafish in a novel tank after repeated exposures to chemical alarm cues.

Incomplete or partial information regarding predation risk may induce uncertainty in prey [reviewed by (Crane et al. 2023)]. Our present study repeatedly exposed zebrafish to risk (conspecific alarm cues) without any predator cue during the background risk exposure phase. This lack of information about predation risk may have induced uncertainty about predator identity in zebrafish. When uncertain about the risk associated with a novel environment or cue, prey should exhibit neophobia (Elvidge et al. 2016). Although this neophobic response can incur significant energy costs, it can help the prey avoid risking its life in an unknown environment or cue (Ferrari et al. 2015). For instance, fathead minnows, *Pimephales promelas*, repeatedly exposed to chemical alarm cues without any predator cues showed neophobic predator responses in a novel environment. In experiment 1, when high-risk individuals were exposed to a novel tank, they showed anxiety-like responses to avoid unknown threats. Similarly, when high-risk individuals were exposed to a novel predator odour, they showed neophobic antipredator responses.

Our results suggest that repeated chemical alarm cues exposures reduced intra-session habituation to a novel environment. In our novel tank experiment (Experiment 1), low-risk individuals exhibited an increase in total distance travelled during the latter half of the observation period compared to the first half. In contrast, high-risk individuals did not show any difference in total distance travelled between the first and the latter half of the observation period. However, high-risk and low-risk individuals showed a significant reduction in time spent in the bottom zone during the last four minutes compared to the first four. At least for the total time travelled, these findings align with Wong et al. (2010), who reported that exposure to alarm cues in a novel tank weakened habituation in zebrafish. Our studies provide evidence that a high predation risk background can reduce spatial working memory in a novel environment. This could result in poorer spatial learning performance by high-risk prey. For instance, Braithwaite and Brown (2004) found that the high-risk population of a tropical poecilid, *Brachyraphis episcopi*, performed poorly in a spatial learning task. In our ever-changing world, species often find themselves in novel environments with unfamiliar predators. While exhibiting a neophobic or anxiety-like responses can undoubtedly confer survival benefits to prey, the ‘maladaptive defensive carry-over’ concept suggests that an abrupt shift from a high-risk to a low-risk environment can result in unnecessary energy expenditure related to anxiety-like behaviours (Crane et al. 2020). The slower habituation observed in high-risk individuals in the novel tank (a non-risky environment) compared to the low-risk counterparts provides evidence of a short-term ‘maladaptive carry-over effect’.

Interestingly, in Experiment 2, within a short time (30 min after introduction to the novel tank), there was no significant difference in anxiety-like behaviours between the high-risk and the low-risk individuals. There are three possible reasons for this. First, individuals may have stopped exhibiting anxiety-like behaviors shortly after the initial introduction due to the lack of negative reinforcement in the novel tank. Brown et al. (2015) reported that repeated exposure to a novel odour without negative reinforcement allowed juvenile convict cichlids (*Amatitlania nigrofasciata*) to reduce their neophobic responses quickly. Second, zebrafish are known for their robust habituation ability (Wong et al. 2010). Lastly, the zebrafish population we used may generally be bolder and showed more risk-taking behaviour in the novel tank. The absence of negative reinforcement, combined with a strong habituation ability and a greater tendency for risk-taking, may have led to similar baseline anxiety responses between the low-risk and the high-risk individuals before the novel odor exposure in the novel tank after 30 min. It suggests that neophobia is a plastic response: individuals initially display anxiety-like or fear behaviors in new environments or in response to novel stimuli, but as they learn that these stimuli are not threats, they cease to show costly fear responses. Similarly, high-risk individuals showed an increase in distance covered during the last 3 min compared to the first 3 min following the novel predator odour exposure. However, we did not record when zebrafish stopped exhibiting fear responses following the novel odour exposure. Future studies may compare the duration of anxiety-like or fear responses between a novel environment and a novel cue.

It is important to interpret the results of Experiment 2 with some caution. The groups in terms of background risk exposures were not homogeneous, with four groups consisting of six individuals and three groups consisting of five. Shishis et al. (2022) reported that tank size and housing density had significant additive and interactive effects on swim path parameters, such as immobility, swim speed, turn angle, and distance to the bottom and stimulus. To account for this, we included exposure tank numbers as a random nesting factor in our analysis to assess any potential impact of varying group sizes. However, no significant differences were observed. This may be due to the limited variability in housing density and uniform tank size across the experiment (i.e., only a one-individual difference), which could have minimized the behavioral deviations caused by zebrafish group size difference.

Future studies should examine whether abrupt shift from a high-risk to a low-risk environment would result in unnecessary energy expenditure due to high-risk phenotypic responses. Only a few studies have investigated the role of background risk on prey’s learning performance other than predator related information (Braithwaite and Brown 2004; Burns and Rodd 2008; Guido et al. 2017). More studies on the effects of neophobia on spatial and latent learning will allow us to explore the influence of predation pressure in prey. It will help researchers to predict which species will be well-adaptive or more vulnerable in the face of habitat shift.

## Electronic supplementary material

Below is the link to the electronic supplementary material.

Supplementary Material 1

Supplementary Material 2

## Data Availability

Behavioural data that support the findings of this study is provided as a supplementary information files.
