# Supplementary figures and images for: Background predation risk induces anxiety-like behaviour and predator neophobia in zebrafish
Source: Anim Cogn. 2024 Oct 23;27(1):69. doi: 10.1007/s10071-024-01908-z (PMC11499451; doi:10.1007/s10071-024-01908-z)

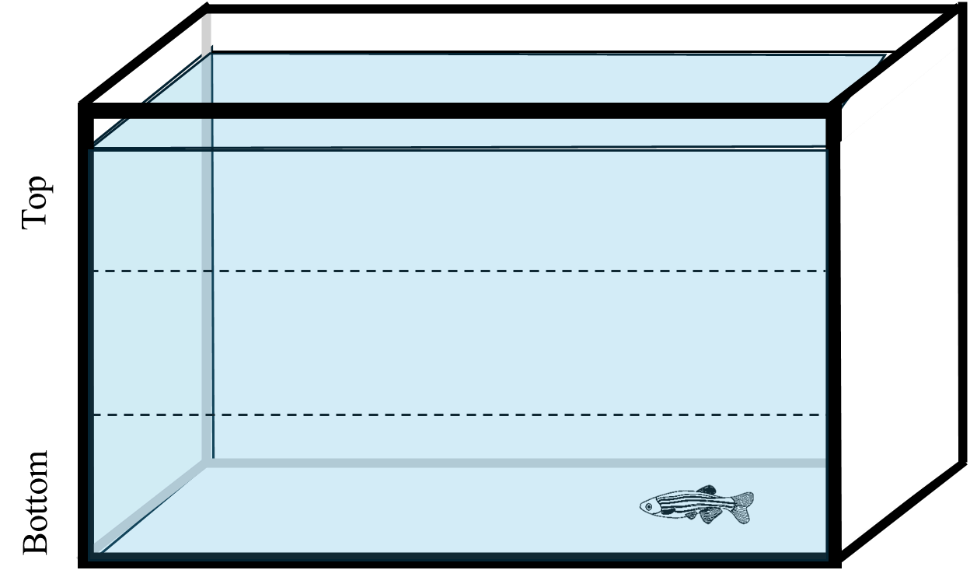


Fig. S1 Depiction of a novel tank with different zones indicated

Supplement: Supplementary file 1 — Supplementary Material 1 [file 10071_2024_1908_MOESM1_ESM.docx]
